# KRAS mutation-selective requirement for ACSS2 in colorectal adenoma formation

**DOI:** 10.21203/rs.3.rs-3931415/v1

**Published:** 2024-02-22

**Authors:** Konstantin Budyagan, Alexa C. Cannon, Adam Chatoff, Nathaniel W. Snyder, Alison M. Kurimchak, James S. Duncan, Jonathan Chernoff

**Affiliations:** 1 Department of Biochemistry & Molecular Biology, Drexel University College of Medicine, Philadelphia, Pennsylvania, United States of America; 2 Department of Cancer & Cellular Biology, Lewis Katz School of Medicine, Temple University, Philadelphia, Pennsylvania, United States of America; 3 Cancer Signaling & Microenvironment Program, Fox Chase Cancer Center, Philadelphia, Pennsylvania, United States of America

## Abstract

Oncogenic *KRAS* mutations are prevalent in colorectal cancer (CRC) and are associated with poor prognosis and resistance to therapy. There is a substantial diversity of *KRAS* mutant alleles observed in CRC. Emerging clinical and experimental analysis of common *KRAS* mutations suggest that each mutation differently influences the clinical properties of a disease and response to therapy. Although there is some evidence to suggest biological differences between mutant *KRAS* alleles, these are yet to be fully elucidated. One approach to study allelic variation involves the use of isogenic cell lines that express different endogenous *Kras* mutants. Here, we generated *Kras* isogenic *Apc*^*−/−*^ mouse colon epithelial cell lines using CRISPR-driven genome editing by altering the original G12D *Kras* allele to G12V, G12R, or G13D. We utilized these cell lines to perform transcriptomic and proteomic analysis to compare different signaling properties between these mutants. Both screens indicate significant differences in pathways relating to cholesterol and lipid regulation that we validated with targeted metabolomic measurements and isotope tracing. We found that these processes are upregulated in G12V lines through increased expression of nuclear SREBP1 and higher activation of mTORC1. G12V cells showed higher expression of ACSS2 and ACSS2 inhibition sensitized G12V cells to MEK inhibition. Finally, we found that ACSS2 plays a crucial role early in the development of G12V mutant tumors, in contrast to G12D mutant tumors. These observations highlight differences between KRAS mutant cell lines in their signaling properties. Further exploration of these pathways may prove to be valuable for understanding how specific KRAS mutants function, and identification of novel therapeutic opportunities in CRC.

## INTRODUCTION

Colorectal cancer (CRC) is one of the most common malignancies worldwide^1^. *KRAS* mutations are observed in about 45% of CRC cases and are clinically associated with tumor invasion and metastasis, resistance to therapy, and a worse overall survival^2–4^. The most common sites of oncogenic mutations of *KRAS* are located at codons 12, 13, 61, 117, and 146. Unlike other cancers that are associated with KRAS mutations, CRC is unique for its diversity of *KRAS* alleles, with the most common being the G12D (28%), G12V (20%), and G13D (16%)^4,5^. Like other small GTPases, KRAS acts as binary molecular switches that cycle between an active GTP-bound state and an inactive GDP-bound state. Guanine nucleotide exchange factors (GEFs), such as SOS and RasGRP, assist in the exchange of GDP to GTP^6–8^. GTPase activating proteins (GAPs) such as NF1 or p120GAP mediate GTP hydrolysis and help inactivate RAS^9,10^. Activating mutations in the *KRAS* result in aberrant Ras signaling due to an altered balance of the active and inactive forms, by reducing GTP hydrolysis or by increasing the rate of GTP loading. In this active state, Ras activates many downstream effector pathways such as MAPK and PI3K-AKT signaling pathway, responsible for cell cycle progression, protein synthesis, pro-growth metabolism, and overall cell survival.

Recent studies have extensively documented differences in the biochemical and signaling properties of common KRAS variants^11–14^. For example, both G12D and G12V mutations have reduced affinity for RAF effector, however, the G12V mutation is predicted to activate RAF better due to its reduced GTPase activity^12^. Distinct mutant-specific properties suggest that each mutation can differently influence the prognosis of disease and response to therapy. For example, among the most common KRAS mutations found in CRC, G12V is the only associated with worse overall survival^15,16^. Although there is evidence to suggest biological differences between mutant G12V vs other *KRAS* alleles, these mechanistic distinctions remain unclear.

Recent efforts to target KRAS directly have led to the FDA approval of allele specific KRAS^G12C^ inhibitor, however, G12C mutations only account for ~3% of CRC cases^17^. Furthermore, unlike in non-small cell lung cancer (NSCLC), the initial response rates to G12C inhibitors have been poor in colorectal cancer^18,19^. Potential resistance mechanisms include upstream activations of several receptor tyrosine kinases and faster signaling rebound, highlighting the challenges of targeting KRAS mutant CRC^20^. The development of other mutant-specific as well as pan-KRAS inhibitors is promising, however given the limited clinical outcome of G12C inhibitors in CRC, these agents will mostly likely need to be administered in combination with other inhibitors. Therefore, full understanding of different biological properties of the mutant KRAS alleles that contribute to their clinical properties is essential for the development of a successful allele-specific therapeutic intervention.

In this study, we explore the signaling differences between KRAS alleles using *Kras* isogenic *Apc*^*−/−*^ mouse colon epithelial cell lines. These cell lines were generated using CRISPR-driven genome editing by altering the original G12D *Kras* allele to G12V, G12R, or G13D. The ultimate focus is to investigate unique properties of G12V relative to other common KRAS mutations found in CRC. These cell lines were utilized to perform transcriptomic and proteomic analyses to compare different signaling properties between mutants. Both analyses indicate significant differences in pathways relating to cholesterol and lipid regulation. We found that these processes are upregulated in G12V lines through increased expression of nuclear SREBP1 and higher activation of mTORC1. Furthermore, G12V cells showed greater acetate utilization, increased expression of ACSS2, and hyperacetylation of RAPTOR. Inhibition of ACSS2 preferentially sensitized G12V cells to MEK inhibitors. Finally, we show that ACSS2 is necessary for the formation of G12V mutant tumors after engraftment into mice. These observations suggest that ACSS2 plays a crucial role early in the development of G12V mutant tumors, and may further facilitate their proliferation by utilizing acetate as a carbon source for lipogenesis and reducing their sensitivity to targeted therapies. Targeting ACSS2 may be a promising therapeutic approach to be a part of a combination therapy to target G12V mutant CRC.

## RESULTS

### Signaling differences between Kras^WT/G12D^, Kras^WT/G12V^, Kras^WT/G12R^, and Kras^WT/G13D^ mutations in isogenic mouse colon epithelial cells.

**Figure 1.**
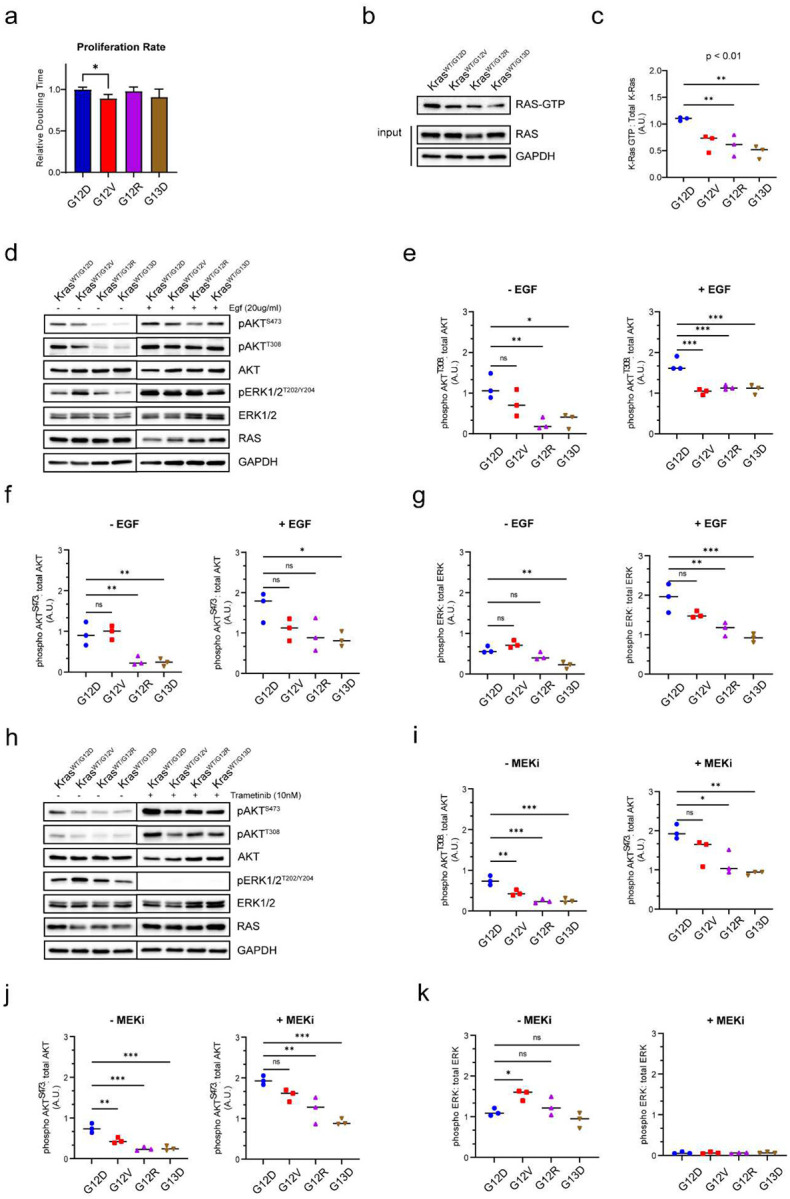
Signaling differences between *Kras*^*WT/G12D*^, *Kras*^*WT/G12V*^, *Kras*^*WT/G12R*^, and *Kras*^*WT/G13D*^ mutations in mouse colon epithelial cells. **A**. Relative doubling time of *Kras*^*MUT*^ cells measured by xCELLigence RTCA. **B**. Representative blot for affinity purification of active Ras using Raf-RBD. 500ug of lysate was used for the pulldown and 25ug for total protein. Results are representative of three similar experiments. **C**. Quantification of band intensities in **B**. One-way ANOVA, ** p<0.01. **D**. Representative western blot for activation of downstream signaling in *Kras*^*MUT*^ mouse colon epithelial cells cultured in 0.5% FBS and upon stimulation with EGF for 20 min. Results are representative of three similar experiments. **E-G**. Quantification of western band intensities in **D**., for p-Akt^T308^**(E)**, p-Akt^S473^**(F)**, and p-Erk **(G)**. One way ANOVA, ns not significant, * p<0.05, ** p<0.01, *** p<0.001, *** p<0.0001. **H**. Representative western blot for downstream signaling in *Kras*^*MUT*^ mouse colon epithelial cells in response to 24hr MEK inhibition (10nM Trametinib). Results are representative of three similar experiments. **I-K**. Quantification of western band intensities in **H** for p-Akt^T308^**(I)**, p-Akt^S473^**(J)**, and p-Erk **(K)**. One way ANOVA, ns not significant, * p<0.05, ** p<0.01, *** p<0.001, *** p<0.0001.

The role of mutant *KRAS* alleles on cellular proliferation, downstream signaling, and sensitivity to inhibitors of downstream effectors was evaluated in the mouse colon epithelial isogenic cell line system. Proliferation was assessed using the xCELLigence RTCA system, which was used to calculate the doubling time during logarithmic growth. Subtle differences in proliferation were noted between the cell lines carrying each of the different *Kras* mutant alleles, with G12V expressing cells showing a slightly lower doubling time (Fig. 1A), suggesting that mutant *Kras* alleles can have a subtle effect on cellular proliferation in this cell line system.

Next, we investigated the allelic influence of KRAS activation in mouse colon epithelial cells by immuno-precipitation with the RAS binding domain of RAF1 (Raf-RBD). Our results showed that *Kras*^*WT/G12D*^ cells had the highest level of GTP-bound KRAS, indicating a higher level of KRAS activation compared to the other mutants. *Kras*^*Wt/G12R*^ and *Kras*^*WT/G13D*^ had significantly lower levels of GTP-bound KRAS relative to *Kras*^*WT/G12D*^. Interestingly, *Kras*^*WT/G12V*^ cells had slightly lower level of active KRAS than *Kras*^*WT/G12D*^, however it was not significant (Fig. 1B,C).

To assess the allelic consequences on downstream signaling, we evaluated RAF and PI3K pathway activation. Phosphorylation of AKT indicates PI3K pathway activation and ERK phosphorylation indicates RAF activation^21,22^. In low serum culture conditions, G12D and G12V cells displayed clear activation of the downstream signaling effector AKT, whereas G12R and G13D cells showed lower levels of phospho-AKT (pAKT^S473^/pAKT^T308^) (Fig. 1D–F). All mutants showed ERK activation, however G13D cells showed lower levels relative to the other cell lines. All KRAS mouse colon epithelial cells remained responsive to upstream mitogenic signals, as acute EGF stimulation increased ERK activity and AKT phosphorylation in all mutants (Fig. 1D–G).

**Figure 2.**
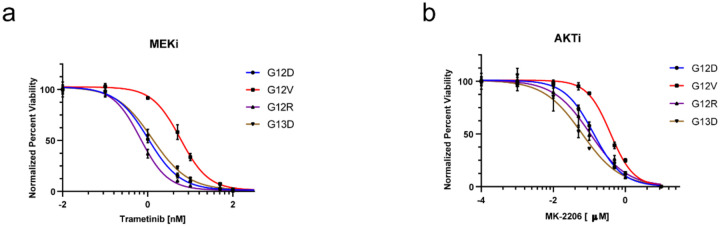
Sensitivity for *Kras*^*WT/MUT*^ mouse colon epithelial cells to inhibitors of downstream effector pathways. **A-B**. Response of *Kras*^*WT/MUT*^ mouse colon epithelial cells to MEK inhibition (Trametinib) **(A)**, and AKT inhibition (MK2206) **(B)**. Cells were cultured at various doses of inhibitor for 72hr. Viability was evaluated using Cell-Titer Glo luminescence. Representatives results from three biological experiments per genotype are shown, curves were fit with nonlinear regression in GraphPad Prism 9.

In response to a MEK inhibitor (Trametinib), all KRAS mutants responded with increased AKT activation (Fig. 2A–D), consistent with previous studies highlighting PI3K/AKT pathway as a major mechanism of resistance to MEK inhibition^23,24^. To further assess the dependency on downstream effectors, KRAS mutant cells were evaluated for their sensitivity to selective inhibitors of AKT and MEK. Interestingly, the sensitivity profiles were different among mutants, with G12V cells showing the lowest sensitivity to both inhibitors (Fig. 2E,F). Overall, these data show that downstream effects of individual KRAS mutations differ in the mouse colon epithelial cell line model.

### Interrogation of global expression changes in KRAS mutant expressing mouse colon epithelial cells by proteogenomic analysis

To gain deeper analysis of allele-specific signaling, we performed global transcriptomics using RNA-sequencing on mouse colon epithelial cells expressing *Kras*^*WT/G12D*^, *Kras*^*WT/G12V*^, *Kras*^*WT/G12R*^, and *Kras*^*WT/G13D*^, respectively. RNA extraction and quality control were performed on all samples together, followed by library preparation and mRNA sequencing at the Next Generation Sequencing Facility at Fox Chase Cancer Center.

Principal component analysis (PCA) of data revealed that allele replicates clustered together, but that different genotypes separated in principal component space. Samples expressing G12R tended to cluster closer to those expressing G13D than to those expressing G12D or G12V. G12D and G12V formed separate clusters, indicating a clear distinction in the global gene expression profiles of the two alleles (Supplemental Fig. 1).

**Figure 3.**
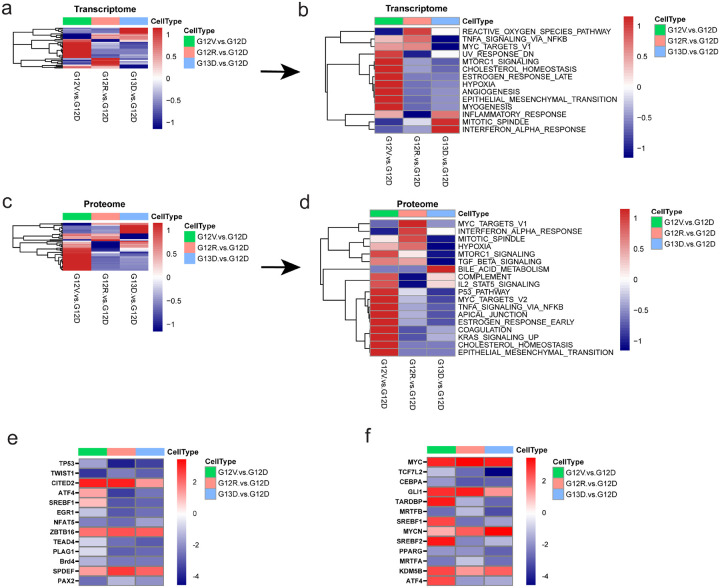
Global proteomics and transcriptomics analysis of *Kras*^*WT/MUT*^ mouse colon epithelial cells. **A, B**. Summarize GSEA results for hallmark gene sets, as determined by bulk RNA sequencing of KRAS mutant murine CRC. **A**. Global view of GSEA results comparing G12V:G12D (denoted by green columns), G12R:G12D (denoted by peach color), and G13D:G12D (denoted by blue columns) B. Detailed heatmap of significantly enriched hallmark gene sets enriched in G12V, G12R, or G13D cells relative to G12D. FDR (false discovery rate) < 0.05, logFC (log fold-change) > 2. **C, D**. Summarize GSEA results for hallmark gene sets, as determined by global proteomic analysis of KRAS mutant cells. **C**. Global view of GSEA results for hallmark gene sets comparing G12V:G12D, G12R:G12D, and G13D:G12D. **D**. Detailed heatmap of significantly enriched hallmark gene sets enriched in G12V, G12R, or G13D cells relative to G12D. FDR (false discover rate) < 0.05, logFC (log fold-change) > 2. **E**. Heatmap showing Ingenuity Pathway Analysis (IPA) of upstream regulators, as determined by bulk RNAsequencing, comparing G12V:G12D, G12R:G12D, and G13D:G12D. Upstream regulators are ranked according to the z-score that predicts activation (Red)/suppression (blue). **F**. Heatmap showing Ingenuity Pathway Analysis (IPA) of upstream regulators, as determined by global proteome analysis, comparing G12V:G12D, G12R:G12D, and G13D:G12D. Upstream regulators are ranked according to the z-score that predicts activation (Red)/suppression (blue). FDR (false discovery rate) <0.2, logFC (log fold change) >1.5.

To gain a pathway-level understanding of the RNA-seq data, we performed a gene set enrichment analysis (GSEA) on mouse colon epithelial cells expressing G12D, G12V, G12R, and G13D mutants. Our goal was to identify hallmark gene sets enriched in each mutant relative to the *Kras*^*WT/G12D*^ original cell line. Several pathways showed significant enrichment or de-enrichment in *Kras*^*WT/G12V*^, *Kras*^*WT/G12R*^, or *Kras*^*WT/G13D*^ relative to *Kras*^*WT/G12D*^ (Fig. 3A). Given the unique properties of the in *Kras*^*WT/G12V*^ cell line, we were particularly interested in identifying unique pathways upregulated/downregulated in *Kras*^*WT/G12V*^ relative to others. Of note, MTORC1_SIGNALING and CHOLESTEROL_HOMEOSTASIS gene sets are uniquely enriched in *Kras*^*WT/G12V*^ cells (Fig. 3B).

Next, we conducted a global proteomic analysis to validate our transcriptomic data. Our aim was to identify overlapping gene sets from proteogenomic analyses enriched in G12V, G12R, and G13D expressing cells compared to G12D, using GSEA analysis. We found that, as in our RNA-seq analysis, the MTORC1_SIGNALING and CHOLESTEROL_HOMEOSTASIS gene sets were consistently enriched in *Kras*^*WT/G12V*^ cells (Fig. 3C,D).

To evaluate the upstream transcriptional regulators responsible for the observed gene expression changes, we performed Ingenuity Pathway Analysis (IPA) of upstream regulators using both transcriptomic and global proteomic data. IPA identified SREBF1, SREBF2, and ATF4 as uniquely activated potential upstream regulators in *Kras*^*WT/G12V*^ cells (Fig. 3E,F). These transcription factors have been previously shown to be regulated by mTORC1^21,25–27^. Together, these results highlight the distinct signaling profiles driven by each mutant KRAS allele, emphasizing that mutant KRAS alleles are not equal. Specifically, KRAS G12V expressing cells have higher mTORC1 activity leading to enrichment in signatures consistent of increased lipogenesis.

### Kras^WT/G12V^ mouse colon epithelial cells exhibit greater acetate utilization to generate cholesterol compared to other mutants.

Given the strong mTORC1 and cholesterol biosynthesis signature observed in *Kras*^*WT/G12V*^ cells, we wanted to explore the pathway further in the context of all the mutant *Kras* alleles. One of the major mechanisms of mTORC1 driven lipogenesis is through regulation of transcription factors sterol regulatory element-binding protein (SREBP). Oncogenic and growth factor signaling in cancer cells increases mTORC1 signaling, resulting in translocation of SREBPs from the endoplasmic reticulum to the nucleus, where they upregulate genes for *de novo* lipid and cholesterol synthesis^28^. SREBPs play a crucial role in regulating genes encoding ACLY, ACSS2, ACC, among others that drive lipogenesis^25,26,29^.

**Figure 4.**
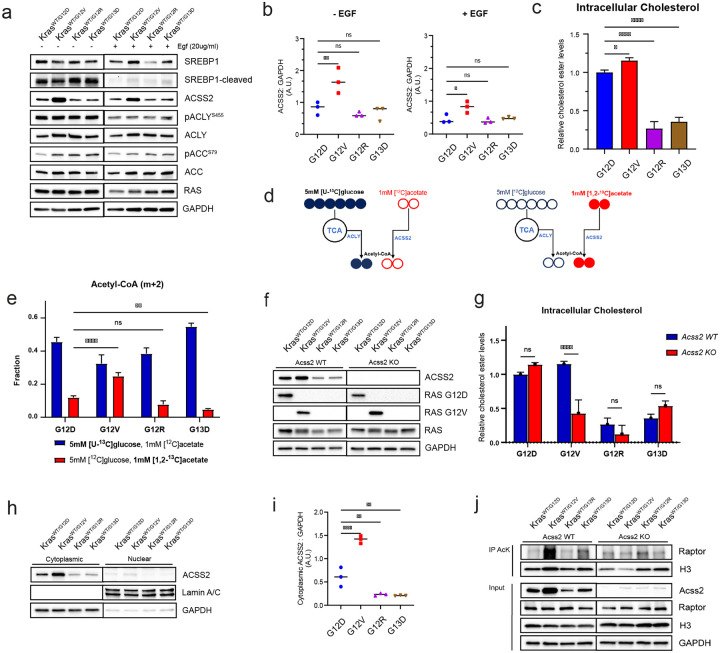
*Kras*^*WT/G12V*^ mouse colon epithelial cells show increased ACSS2 utilization to generate cholesterol. **A**. Representative western blot for expression and activation of lipogenic enzymes in *Kras*^*WT/MUT*^ mouse colon epithelial cells cultured in 0.5% FBS and upon stimulation with EGF for 20 min. Results are representative of three similar experiments. **B**. Quantification of western band intensities in **A**., for ACSS2. One way ANOVA, ns not significant, * p<0.05, ** p<0.01, *** p<0.001, *** p<0.0001. **C**. Intracellular cholesterol ester levels in KRAS mutant cells. Data shows Cholesterol Ester-Glo luminescence averages from three biological replicates. One way ANOVA, ns not significant, * p<0.05, *** p<0.0001. **D**. Experimental design for heavy isotope labeling of acetyl-CoA using [U-^13^C] glucose with unlabeled acetate (left), and [1,2-^13^C] acetate with unlabeled glucose (right). **E**. m+2 acetyl-CoA following 24hr labeling with 5 mM [U-^13^C] glucose with 1 mM unlabeled acetate (left), and 1 mM [1,2-^13^C] acetate with 5 mM unlabeled glucose. **F**. Representative western blot confirming ACSS2 knockout using CRISPR. **G**. Intracellular cholesterol ester levels in KRAS mutant cells with ACSS2 KO. Data shows Cholesterol Ester-Glo luminescence averages from three biological replicates. One way ANOVA, ns not significant, * p<0.05, ***. **H**. Representative western blot of ACSS2 subcellular localization in KRAS mutant cells. **I**. Quantification of western band intensities in **H**, for cytoplasmic ACSS2. One way ANOVA, ** p<0.01, *** p<0.0001. **J**. ACSS2-dependent acetylation of Raptor in KRAS-G12V CRC cells. Proteins were immunoprecipitated with anti-acetylated lysine Abs, and the IPs were probed with anti-Raptor Abs.

To evaluate to influence of KRAS alleles on expression of key genes driving lipogenesis, western blot analysis was performed. In low serum culture conditions, all four mutants exhibited increased cleaved SREBP1, indicating a nuclear fraction that drives lipogenesis. Cytoplasmic acetyl-CoA is a central metabolic intermediate that acts as a precursor for lipogenesis. During states where acetyl-CoA is needed, citrate is the predominant source of acetyl-CoA, when it is cleaved by ATP-citrate lyase (ACLY) to generate oxaloacetate and acetyl-CoA^30^. An alternative route utilizes of acetyl-CoA synthetase short-chain family member 2 (ACSS2) to convert cytoplasmic acetate to acetyl-CoA^31,32^. There were no significant differences observed in phosphorylation of ACLY at its activation site, S455, among the mutants. In contrast, the expression of ACSS2 was significantly elevated in G12V cells under low serum conditions and remained elevated after growth factor stimulation (Fig. 4A,B). These findings suggest that ACSS2 may be a crucial contributor to the elevated lipogenesis observed in *Kras*^*WT/G12V*^ mutant cells. To confirm elevated expression of *Acss2* in G12V mutant cells, qPCR analysis was performed comparing expression levels in three different G12V clones relative to starting G12D cell line. All three G12V clones showed >2-fold increase in *Acss2* expression relative to G12D (Supplemental Fig. 2).

To investigate the relationship between ACSS2 expression and intracellular cholesterol levels in colorectal cancer, we measured cholesterol ester levels in KRAS mutant mouse colon epithelial cells. Interestingly, G12D and G12V expressing cells exhibited significantly elevated cholesterol levels compared to G12R and G13D cells, with G12V having the highest levels (Fig. 4C). These results suggest that the G12D and G12V mutations may play a more significant role in the regulation of cholesterol metabolism than the G12R and G13D mutations in colorectal cancer.

Elevated levels of ACSS2 in *Kras*^*WT/G12V*^ cells relative to other mutants, suggest an increased utilization of acetate to generate cytoplasmic acetyl-CoA for lipogenesis in those cells. To determine the respective use of glucose- and acetate-derived carbon for cytoplasmic acetyl-CoA generation, we carried out stable isotope tracer experiments. KRAS mutant cells were incubated with 5 mM [U-^13^C] glucose and 1 mM unlabeled acetate, or reciprocally, 1 mM [1,2-^13^C] acetate and 5 mM unlabeled glucose (Fig. 4D) for 24 hours. These experiments showed that *Kras*^*WT/G12D*^ cells received about 12 percent of its acetyl-CoA acyl carbons from acetate, *Kras*^*WT/G12R*^ received about 10 percent from acetate, while KRAS^WTG13D^ received about 5 percent from acetate. Interestingly, *Kras*^*WT/G12V*^ cells received about 25% of acetyl-CoA acyl carbons from exogenous acetate, significantly higher than other mutants, indicating greater utilization of acetate at normal culture conditions. *Kras*^*WT/G13D*^ were also unique as they received about 95 percent of acetyl-CoA acyl carbons from glucose (Fig. 4E).

To further investigate the importance of ACSS2 in KRAS mutant mouse colon epithelial cells, we generated ACSS2 knockout cells using CRISPR-mediated gene knockout (Fig. 4F). Intracellular cholesterol levels were then evaluated in ACSS2 WT and KO cells. Interestingly, *Kras*^*WT/G12V*^; *Acss2 KO* cells showed significantly decreased levels of cholesterol relative to *Kras*^*WT/G12V*^; *Acss2 WT*. Cholesterol levels in other KRAS mutant cells were not significantly impacted in ACSS2-KO cells (Fig. 4G). These findings suggest that *Kras*^*WT/G12V*^ cells have greater expression of ACSS2 which is localized to the cytoplasm, that such cells exhibit greater utilization of acetate for lipogenesis at normal conditions, and that ACSS2 knockout uniquely affects G12V mutants, compromising their ability to generate intracellular cholesterol.

### ACSS2-dependent hyperacetylation of RAPTOR in KRAS G12V cells.

ACSS2 is present in both the nucleus and cytoplasm of cells. While cytosolic ACSS2 plays a critical role in the de novo biosynthesis of lipids, nuclear ACSS2 is involved in the recycling of acetate produced from histone deacetylation reactions^33–35^. To investigate the localization of ACSS2 in KRAS mutant mouse colon epithelial cells, we fractionated the cells into cytoplasmic and nuclear fractions and immunoblotted for ACSS2. This experiment indicated that ACSS2 was primarily localized in the cytoplasmic fraction, with G12V mutations showing consistently higher expression levels (~2-fold higher) compared to G12D mutations, and up to ~7-fold higher expression levels than G12R and G13D mutations (Fig. 4H,I). These findings suggest that different KRAS mutations may lead to distinct metabolic changes in colorectal cancer cells, with G12V mutations potentially having a greater impact on lipid metabolism due to higher ACSS2 expression levels.

While our data point to cholesterol synthesis as a key recipient for acetate-produced acetyl-CoA in KRAS^G12V^ CRC cells, it is also possible that this metabolite affects MEKi sensitivity because it promotes histone acetylation, with subsequent changes in gene regulation^39^. However, as ACSS2 is primarily cytosolic in KRAS^G12V^ cells (Fig. 4H,I), non-histone targets seem more likely. We therefore considered potential cytosolic targets of acetylation. Son *et al*. recently reported that the mTORC1 component RAPTOR is activated by acetylation on K1097^33^. Intriguingly, we found that RAPTOR is heavily acetylated only in KRAS G12V cells, and that this acetylation is dependent on ACSS2 (Fig. 4J). This observation suggests a unique molecular link between ACSS2, mTORC1, and lipid synthesis in KRAS G12V cells.

### Inhibition of ACSS2 in KRAS G12V mouse colon epithelial cells sensitizes them to MEK inhibition.

**Figure 5.**
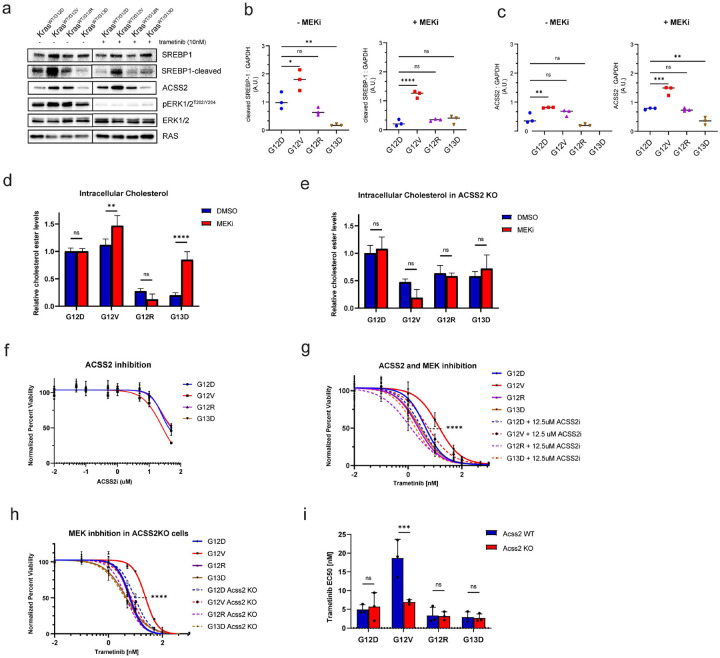
Inhibition of ACSS2 sensitizes *Kras*^*WT/G12VT*^ mouse colon epithelial cells to MEK inhibition. **A**. Representative western blot for SREBP-1 and ACSS2 in *Kras*^*MUT*^ mouse colon epithelial cells in response to 24hr MEK inhibition (10nM Trametinib). Results are representative of three similar experiments. **B-C**. Quantification of western band intensities in **A** for cleaved SREBP-1 **(B)**, and ACSS2 **(C)**. One way ANOVA, ns not significant, * p<0.05, ** p<0.01, *** p<0.001, *** p<0.0001. **D**. Sensitivity of KRAS mutant mouse colon epithelial cells to ACSS2 inhibitor. **E**. Sensitivity of KRAS mutant mouse colon epithelial cells to MEK inhibition (trametinib) in combination with an ACSS2 inhibitor. **F**. Sensitivity of KRAS mutant, ACSS2 KO mouse colon epithelial cells to MEK inhibition (trametinib). **D-E**. Data shows Cell-Titer Glo luminescence averages, curves were fit with nonlinear regression in GraphPad Prism 9, extra sum of squares f test was used to compare difference in logIC50. **** p<0.0001. **G**. EC50 values of trametinib sensitivity for each biological replicate, as calculated in GraphPad Prism, were plotted by mutant and ACSS2 status. One-way ANOVA, ns not significant, *** p<0.001. **H**. Intracellular cholesterol ester levels in KRAS mutant cells in response to 24hr MEK inhibition. Data shows Cholesterol Ester-Glo luminescence averages from three replicates. One way ANOVA, ns not significant, * p<0.05, *** p<0.0001 **I**. Intracellular cholesterol ester levels in *Kras*^*WT/MUT*^, *Acss2 KO* mouse colon epithelial cells in response to 24hr MEK inhibition. Data shows Cholesterol Ester-Glo luminescence averages from three replicates. One way ANOVA, ns not significant.

The RAF-MEK-ERK pathway is the canonical pathway downstream of KRAS^34,35^ and numerous studies have highlighted the key role of MAPK pathway in driving KRAS mutant-dependent growth^36^. To evaluate the effect of MEK inhibition on the expression of SREBP1 and ACSS2, we performed an immunoblot analysis. G12V cells had higher levels of nuclear SREBP compared to other mutants, consistent with the proteogenomic data. After 24 hours of MEK inhibition, the levels of SREBP decreased in G12D, G12R, and G13D mutant cells, but remained elevated in G12V cells (Fig. 5A,B). This observation suggests that MEK inhibition has a varied effect in KRAS mutants in context of SREBP expression, possibly due to distinct downstream signaling mechanisms. Furthermore, both *Kras*^*WT/G12D*^ and *Kras*^*WT/G12V*^ showed significantly increased expression of ACSS2 in response to MEK inhibition; however *Kras*^*WT/G12V*^ mutant cells maintained the highest expression level compared to other mutants, ~2 fold higher that G12D cells (Fig. 5A,C).

Given this increased ACSS2 expression in G12V cells in response to MEK inhibition, we investigated whether this correlated with intracellular cholesterol levels. Intracellular cholesterol ester levels were measured in response to MEK inhibition in KRAS mutant cells. G12D and G12R mutant cells were not altered in response to MEK inhibition, however G12V and G13D mutants exhibited a significant increase in cholesterol levels following trametinib treatment. Interestingly, in ACSS2 KO cells, none of the mutants showed any significant changes in intracellular cholesterol levels upon trametinib treatment (Fig. 5D,E). These results suggest that the G12V mutation displays a unique signaling profile in response to MEK inhibition and may confer a greater dependence on the ACSS2 pathway for cell growth relative to other mutants.

To evaluate the potential dependence of KRAS mutant cells on ACSS2, we assessed the effects of a small molecule inhibitor of ACSS2 on cell viability. While the ACSS2 inhibitor alone had no significant effects, ACSS2 inhibition increased the sensitivity of G12V cells to trametinib, while the sensitivity of other KRAS mutants to trametinib was not altered (Fig. 5F,G). Furthermore, *Kras*^*WT/G12V*^; *Acss2 KO* cells showed significant decrease in sensitivity to trametinib compared to *Kras*^*WT/G12V*^*; Acss2 WT*, however knockout of ACSS2 did not alter other mutants’ sensitivity to MEK inhibition, as indicated by their calculated EC50 values (Fig. 5H,I). Taken together, these data suggest that *Kras*^*WT/G12V*^ mouse colon epithelial cells are particularly dependent on ACSS2 for survival, which affects their sensitivity to MEK inhibition, rendering them particularly sensitive to ACSS2 inhibition in combination with trametinib.

### Dual inhibition of ACSS2 and MEK in human isogenic cells lines selectively targets KRAS^G12V^ cells.

Our results suggest the potential utility of targeting ACSS2 in *Kras*^*WT/G12V*^ colorectal adenocarcinoma, which could have implications for the development of new cancer therapies. To determine if the same dependency is evident in additional KRAS-mutant, CRC cell lines, we evaluated this therapeutic approach in two isogenic human cell line systems (LIM1215, and SW48) that comprise a series of *KRAS* mutant alleles, similar to the mouse cells.

**Figure 6.**
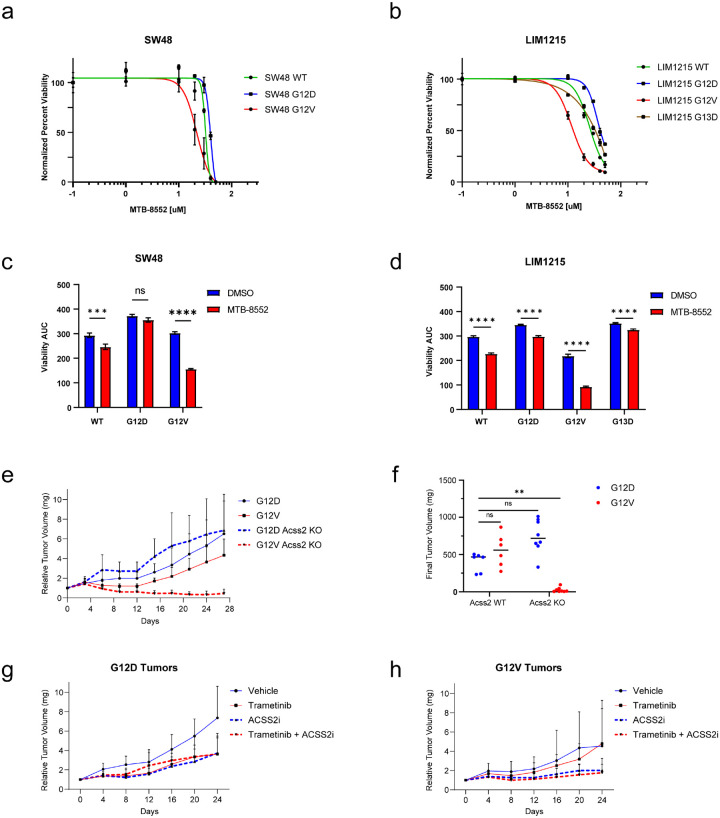
ACSS2 is required for KRAS G12V-driven tumor growth. **A**. Sensitivity of SW48 WT, G12D, and G12V isogenic cell lines system to an ACSS2 inhibitor. **B**. Sensitivity of LIM1215 WT, G12D, G12V, and G13D isogenic cell lines to an ACSS2 inhibitor. **C**. Sensitivity of SW48 isogenic cell lines to MEK inhibition alone or in combination with an ACSS2 inhibitor. Area under the curve (AUC) was calculated in GraphPad Prism. Blue is calculated AUC in response to MEK inhibition alone, red is AUC in response to MEK and ACSS2 inhibition. **D**. Sensitivity of LIM1215 isogenic cell lines to MEK inhibition alone and in combination with an ACSS2 inhibitor. Area under the curve (AUC) based was calculated in GraphPad Prism. Blue is calculated AUC in response to MEK inhibition alone, red is AUC in response to MEK and ACSS2 inhibition. **E**. Relative tumor volume for mice that were allografted with *KrasWT/G12D*, *KrasWT/G12V*, *KrasWT/G12D Acss2 KO*, and *Kras*^*WT/G12V*^ *Acss2 KO* cells. Tumor volumes were measured every 3 days for 28 days. **F**. Final tumor weight at the end of the experiment. Student t-test, ns not significant, ** p< 0.01 **G**. Relative tumor volume for mice that were allografted with (G) *KrasWT/G12D* and (H) *KrasWT/G12V*. Mice were dosed with 0.2 mg/kg trametinib daily p.o. and/or 15 mg/kg ACSS2i every **2 days via i.p**.

Unlike in mouse colon epithelial cell lines, ACSS2 expression was not significantly different between KRAS mutants, nor was it significantly elevated in response to MEK inhibition in human isogenic CRC cell lines (data not shown). However, as with the mouse KRAS cells, both LIM1215 *KRAS*^*G12V/WT*^ and SW48 *KRAS*^*G12V/WT*^ cells were more sensitive to ACSS2 inhibition ((Fig. 6A,B). As with mouse cells, human LIM1215 KRAS G12V cells showed greater sensitivity to ACSS2 inhibition when used in combination with a MEK inhibitor. Similar results were obtained in the SW48 isogenic cell line systems, suggesting a general sensitivity that might be exploited therapeutically (Fig. 6C,D and Supplementary Fig. 3).

### ACSS2 is required for tumor growth of KRAS^G12V^ mouse colon epithelial cells.

The mouse colon epithelial KRAS mutant isogenic cell line system replicates the early stage of the “classic” adenoma-carcinoma sequence responsible for about 80% of CRC cases. The findings using this model system suggest that G12V expressing cells have higher expression of ACSS2 and are particularly more dependent on ACSS2 for their survival. To investigate this hypothesis, we grafted *Kras*^*WT/G12D*^ *Acss2 KO*, and *Kras*^*WT/G12V*^ *Acss2 KO* cells into the flank of C57/BL/6 and then evaluated for their ability to form tumors and to grow. Remarkably, the *Kras*^*WT/G12V*^ Acss2 KO cells were not able to proliferate despite initial tumor formation without ACSS2 (Fig. 6E,F). These data suggest that ACSS2 plays a crucial role early in the development of G12V mutant tumors, it may further facilitate their proliferation by utilizing acetate as a carbon source for lipogenesis and reducing their sensitivity to targeted therapies.

Next, we grafted *Kras*^*WT/G12D*^ *and Kras*^*WT/G12V*^ onto the flank of C57Bl/6 mice and treated with either vehicle, trametinib, ACSS2 inhibitor, or a combination of trametinib plus ACSS2 inhibitor. In the G12D tumors, tumor growth was significantly inhibited by trametinib and an ACSS2 inhibitor, although combination of the two did not add any benefit (Fig. 6G,H). Contrastingly, the G12V tumors did not respond to trametinib alone. However, they ACSS2 inhibition significantly inhibited tumor growth, and addition of trametinib to ACSS2 inhibition further arrested tumor progression. These results further indicate a unique sensitivity of G12V colon epithelial cells to ACSS2 inhibition.

## Discussion

Decades of research on targeting KRAS in cancer has finally removed the “undruggable” tag from mutant KRAS (39). The availability of new KRAS-targeting therapeutic agents has been accompanied by an increased appreciation that not all KRAS mutations are created equal. Understanding the biological differences between the various mutant alleles can aid in the development of even more effective therapeutic strategies for targeting KRAS. In this study, we establish ACSS2 as a unique metabolic regulator in KRAS G12V mutant cells. Our findings further contribute to the growing evidence that not all KRAS mutants illicit the same biological effects and will benefit from the same therapeutic regimen.

In this study, we evaluate the signaling and functional differences caused by different KRAS mutations in mouse colon epithelial cells. To do so, we utilized the newly generated isogenic *Apc*^*−/−*^ cell line system with endogenous expression of *Kras*^*WT/G12D*^, *Kras*^*WT/G12V*^, *Kras*^*WT/G12R*^, and *Kras*^*WT/G13D*^, with an ultimate focus to investigate unique properties caused by the G12V mutation. Through proteogenomic and expression analysis, we demonstrate that G12V cells show higher levels of cytoplasmic ACSS2 compared to other mutants. ACSS2 utilizes acetate as a carbon source to generate cytoplasmic acetyl-CoA, an essential metabolic precursor for synthesis of sterols and fatty acids. We further show that the G12V mutants exhibit greater acetate incorporation into cytoplasmic acetyl-CoAs. Furthermore, *Acss2* knockout significantly impacted the ability of KRAS^G12V^ mutants to generate cholesterol esters, while having no effect on other KRAS mutants. These effects might be mediated by ACSS2-mediated hyperacetylation of Raptor in KRAS G12V cells, activating mTORC1 (Fig. 4j and Ref^33^). In addition, either genetic knockout or pharmacologic inhibition of ACSS2 further sensitized the G12V mutants to MEK inhibition *in vitro*. Finally, and most remarkably, we show that G12V mutants without ACSS2 are unable to form tumors in mice, whereas G12D mutants were still able to form and growth tumors despite ACSS2 KO, while ACSS2 inhibition *in vivo* results in a significant reduction in tumor growth combined with MEK inhibition. These findings demonstrate unique signaling properties driven by specific KRAS mutations in CRC that have the potential to be exploited for therapeutic benefit.

Acetate is one of the three major short chain fatty acids (SCFAs) produced microbial fermentation in the gut and is therefore available abundantly in the colon and rectum. In fact, microbiota are the main source of acetate within the human body^37^. Although normal acetate levels are associated with protective function for the colon epithelium, elevated acetate levels have been associated with CRC in humans^38–40^. Given the findings in this study, the microbial environment in the colon may provide favorable conditions for KRAS^G12V^ adenomas to arise. Previous studies have highlighted that ACSS2 is needed for fatty acid synthesis when cancer cells proliferate under stressful conditions such as hypoxia^41^. The healthy mucosa of large intestine is already under physiologic hypoxic conditions and as the tumors form and grow, the hypoxic environment is only further exacerbated^42,43^. Therefore, increased ACSS2 expression in G12V cells and their inherent increased utilization of acetate may favor KRAS^G12V^ adenoma formation and continuous growth in the hypoxic colorectal environment. These data suggest that targeting ACSS2 early in the KRAS^G12V^ adenoma formation should inhibit its ability to proliferate and may be a viable therapeutic option as part of an early combination therapy intervention. ACSS2 small molecule inhibitors have been described and one of these is currently in human clinical trials (NCT04990739) in patients with locally advanced or metastatic solid tumors.

A limitation to this study is that KRAS mutant cells were grafted into mice as flank tumors, differing from physiological conditions of the colon. Therefore, tumor proliferation and sensitivity to inhibitors *in vivo* may vary depending on the location of the tumor and the microenvironment. An alternative approach would be to inject the tumors orthotopically^44,45^. This would allow for the evaluation of the effect of ACSS2 in KRAS mutant cells under physiological concentration of acetate in the gut. In addition, these ideas could be tested in GEMM model, *e.g*., mice bearing *Apc*^*fl/fl*^*; Kras*^*Mut/+*^*; Acss2*^*−/−*^ in colonic epithelial cells. This would better address the importance of ACSS2 in KRAS^G12V^ mutant cells versus other mutations and whether it plays a detrimental role for tumorigenesis, similarly as it did in flank tumors.

Another important consideration about the results from this study is that the cell line model used is representative of an early-stage adenoma (*Apc*^*−/−*^*, Kras*^*MUT/WT*^). These results suggest that ACSS2 and an increased lipid metabolism plays an important role early in the G12V mutant cells on their ability to form tumors and sensitivity to targeted inhibitors. One limitation of this study is that the results are based only on studies done in mouse colon epithelial cells. However, there is some evidence to suggest that this may be also true in lung cancers. Recent association study of KRAS genotype and clinicopathologic findings of early-resected NSCLC tumors suggested that G12V genotype was closely associated with enhanced fatty acid and amino acid metabolism^46^. Although not explicitly stated in the study, both of those pathways are consistent with mTORC1 activity. Studies investigating the influence of specific KRAS mutations in patients with early-stage adenoma would further raise our understanding on the specific role of KRAS mutations on the pathogenesis of CRC. A possible experiment of interest to confirm findings in a more relevant system would be to generate human colonic organoids that are *APC* deleted and heterozygous for *KRAS* mutations, similar to the mouse isogenic system. These organoids could then be utilized to evaluate whether G12V mutant cells have higher mTORC1 signaling, SREBP and ACSS2 expression, and greater acetate. This experiment would address whether there is any species-specific differences that might have affected the results using mouse cell lines.

It is possible that as the adenoma progresses to carcinoma and acquires more mutations, these will further alter ACSS2 expression and sensitivity to its inhibition. P53 mutations are found in 50–75% of CRC and occur at later stages of tumor progression^47^. Whether differences in ACSS2 expression and acetate utilization between KRAS mutations will be maintained when p53 mutations are introduced, remains to be determined. P53 plays a regulatory role in lipogenesis and cholesterogenesis by transcriptionally inhibiting the expression of SREBPs^48–50^. Therefore, loss of p53 tumor suppressor may lead to an increased SREBP and ACSS2 expression in all mutants. This might dilute the difference in ACSS2 expression between KRAS mutants and can potentially explain why it has not been reported previously. However, the G12V mutant cells may still maintain their enhanced ability to shift to acetate utilization when stressed, making them less responsive to chemotherapy, thus providing an opportunity to improve treatment by targeting acetate metabolism. Another possibility is that the loss of p53 would make other KRAS mutants more sensitive to ACSS2 inhibition. Whether knockout of p53 in KRAS mutant cells elicits a difference in ACSS2 expression and acetate utilization remains unexplored.

Under basal cell conditions, acetate may not be important for proliferation since during adequate supply of glucose they are able to convert glucose into acetyl-CoA through citrate via ACLY. In contrast, tumor cells, especially when they are becoming increasingly hypoxic and deprived of biosynthetic substrates, require acetate for their proliferation. Therefore, inhibition of ACSS2 should have minimal effect on normally cycling cells, thus making it an attractive target that can be combined with other treatments. In this study, we evaluated the effect of ACSS2 in combination with MEK inhibition. However, despite several clinical trials over the past decade using MEK inhibition in KRAS mutant CRC, as a monotherapy or in combinations with others have not been successful. Therefore, ACSS2 inhibition should be explored with more therapeutically relevant agents. Although there are no G12V specific inhibitors available currently, there are direct and indirect pan-KRAS inhibitors in clinical trials such as SOS-1 inhibitors and RAS(ON) inhibitors respectively.

Colorectal cancer displays the most diverse profile of KRAS mutants with 28% of CRCs harboring a G12D mutation, 20% of CRCs harboring a G12V mutation, and 16% of CRCs harboring a G13D mutation (CITE). Despite the prevalence of CRC worldwide, there has been limited clinical success in treatment of KRAS-driven CRC. While there have been recent breakthroughs in targeting G12C in NSCLC and G12D in pancreatic cancer, there has yet to be a G12V-specific compound and there exists an unmet clinical need for strategies to target this tumor subtype. These data describe ACSS2 as a potential vulnerability in G12V-mutant CRC. Taken together, this work furthers our understanding about differences between KRAS mutations and has established the groundwork for future allele-specific anti-RAS therapies.

## Materials and Methods

### Cell Culture:

C57BL/6 *Apc*^*−/−*^*, Kras*^*WT/G12D*^ cells were gifts from Kevin Haigis (Harvard Medical School). Allelic series of *Kras* mutations were generated by the method described in Chapter II. Cells were cultured in RPMI with 10% FBS, 1% Penicillin/Streptomycin. LIM1215 and SW48 KRAS mutant isogenic cell lines were also gifted by Dr. James Duncan. SW48 were cultured in RPMI with 10% FBS, 1% Penicillin/Streptomycin. LIM1215 were cultured in RPMI (with 2 mM L-Glutamine + 25 mM HEPES) with 10%FBS, 1% Penicillin/Streptomycin, 0.6 μg/mL Insulin, 1 μg/mL Hydrocortisone, 10 μM 1-Thioglycerol. Cells were monitored for mycoplasma every 6 weeks with Universal Mycoplasma Detection Kit (ATCC). All experiments were completed on cells passaged under 20 times.

### xCELLigence proliferation assay:

~ 500 cells/well were seeded into a 16 well PET E-Plate (Agilent technologies) in 100 mL medium. Following seeding and cells were monitored every hour for 120 hours measuring proliferation, attachment, and spreading. Doubling time was calculated by the RTCA software based on the logarithmic phase of the growth curve.

### Active Ras Detection:

Ras-GTP pulldown was performed according to manufacturer’s instruction (Cell Signaling). Briefly, cells were cultured overnight and were harvested at ~80% confluency. Cells were washed with ice-cold PBS and harvested using 0.5 ml Lysis/Binding/Wash Buffer plus 1 mM PMSF (per 10 cm plate) using a scraper into a microcentrifuge tube. The tube was vortexed briefly and incubated on ice for 5 min. Tubes were then microcentrifuged at 16,000 × *g* at 4°C for 15 minutes and supernatant containing cell lysate was transferred to a fresh tube. Protein concentration was measured by BCA protein quantification assay.

Glutathione resin was swirled thoroughly to resuspend the agarose beads, then 100 μL of the 50% resin slurry was added to the spin cup with collection tube. Tubes were spun down at 6,000 × g for 30 sec. Beads were washed using 400uL of 1X Lysis/Binding/Wash buffer and centrifuged at 6,000 × g for 30 sec. 80 μg of GST-Raf-RBD was added to the spin cup containing the glutathione resin, followed by the addition of 500 μg of total protein. The remaining total protein was used for input controls. The mixture was incubated at 4°C for 1 hour with gentle rocking and then spun down at 6,000 × *g* for 30 sec. Resin was washed 3 times by adding 400 μL of 1X Cell Lysis/Binding/Wash Buffer and centrifugation at 6,000 × *g* for 30 sec. 50uL of reducing sample buffer (100 mM DTT in 2X SDS Sample Buffer) was added to the resin, vortexed, incubated for 2 min at room temperature, then spun down at 6,000 × *g* for 2 min. Finally, eluted samples were heated for 5 min at 95°C. 10 μL of eluted sample was use for western blot analysis.

### Western Blotting:

Cells were harvested at about 80% confluency using RIPA buffer with protease (Sigma Aldrich) and phosphatase cocktail inhibitors (Sigma Aldrich). Cells were washed with PBS and incubated with RIPA buffer on ice for 5 minutes. Cells were harvested using a cell scraper and then sonicated with 3 pulses for 10 seconds each at 20% intensity using 550 sonic dismembrator (Fisher Scientific). Cells were centrifuged for 15 minutes at 15,000 × *g* at 4°C and supernatant was transferred to a fresh tube. Protein quantification was performed using BCA protein assay and samples were diluted in sample buffer (1X Laemmli Buffer, 5% 2-mercaptoethanol, BioRad). 20 μg of protein lysate was loaded onto 4–20% gradient Mini-PROTEAN TGX gel (BioRad) and transferred to nitrocellulose membrane for 1 hour. Membranes were blocked using blocking buffer (5% fat-free milk in TBST) for 1 hour at RT then incubated in primary antibodies diluted 1:1000 in blocking buffer overnight at 4°C. Following day, membranes were incubated with secondary antibody at 1:10,000 dilution in blocking buffer and washed 4 times using TBST. Membranes were imaged on FluorChem E (ProteinSimple). Primary antibodies used were GAPDH (Cell Signaling #2118), Ras (Cell Signaling #67648), AKT (Cell Siganling #9272), pAKT^S473^ (Cell Signaling # 4060), pAKT^T308^ (Cell Signaling #13038), pERK (Cell Signaling #4370), ERK (Cell Signaling #4695), AceCS1 (Cell Signaling #3658), pACLY (Cell Signaling #4331), ACLY (Cell Signaling #13390), SREBP (Santa Cruz #sc-13551), pACC (Cell Signaling #11818), ACC (Cell Signaling #4190), Ras-G12D (Cell Signaling #14429), Ras-G12V (Cell Signaling #14412), LAMIN A/C (Cell Signaling #2032). Secondary antibodies used were Peroxidase AffiniPure Goat Anti-Mouse IgG (H+L) (Jackson Lab #115-035-003) and Peroxidase AffiniPure Goat Anti-Rabbit IgG (H+L) (Jackson Lab #111-035-003). Band intensity analysis was performed in ImageJ.

### Cell viability assays:

For cell viability assays, ~1000 mouse colon epithelial cells and ~2500 LIM1215 or SW48 cells were seeded into a white opaque 96-well plate (PerkinElmer). Next day, media was changed to medium supplemented with 2% FBS and inhibitor. Readout of cell proliferation was adopted on cell growth properties avoiding more than 80% confluence in control wells. Cells were then cultured for 72–120 hours depending on the individual doubling times of the cells. Number of living cells were quantified through addition of CellTiter-Glo (Promega) according to the instructions. Plates were read using EnVision 2102 Multilabel Reader (PerkinElmer). EC50 values were calculated in GraphPad Prism 9 based on the point of inflection of a curve.

### RNA sequencing:

Cells were harvested at ~ 80% confluence. Cells were lysed in TRI reagent solution (Invitrogen) and chloroform (Sigma-Aldrich) according to manufacturer’s instructions. Samples were DNase-treated and submitted to the Next Generation Sequencing Facilities at Fox Chase Cancer Center for quality control library, preparation, and paired-end sequencing. Samples were sequenced on an Illumina NextSeq 500 sequencer. Raw data was analyzed by Fox Chase Cancer Center Biostatistics and Bioinformatics Facility.

### Total proteome analysis:

Mouse colon epithelial cells were harvested using lysis buffer (50 mM HEPES pH 8.0, 4% SDS). Protein quantification was performed using BCA protein assay and 100 μg of protein was digested using LysC for 3 hours, followed by trypsin overnight. Following day, digested peptides where isolated using C-18 and PGC columns, then dried and washed using ethyl acetate. Three μg were then resuspended in 0.1% formic acid and separated with a Thermo Scientific RSLC nano Ultimate 3000 LC on a Thermo Scientific Easy-Spray C-18 PepMap 75 mm × 50 cm C-18 2 mm column. A 305 min gradient of 2–20% (180 min) 20%–28% (45 min) 28%–48% (20 min) acetonitrile with 0.1% formic acid was run at 300 nL/min at 50C. Eluted peptides were analyzed by Thermo Scientific Q Exactive or Q Exactive plus mass spectrometers utilizing a top 15 methodology in which the 15 most intense peptide precursor ions were subjected to fragmentation. The AGC for MS1 was set to 3×106 with a max injection time of 120 ms, the AGC for MS2 ions was set to 1×105 with a max injection time of 150 ms, and the dynamic exclusion was set to 90 s. Raw data analysis of LFQ experiments was performed by Fox Chase Cancer Center Biostatistics and Bioinformatics Facility.

### qRT-PCR:

TRI reagent solution (Invitrogen) and chloroform (Sigma-Aldrich) were used to isolate RNA from cells according to manufacturer’s instructions. qRT-PCR on diluted cDNA was performed with inventoried TaqMan Gene Expression Assays on the QuantStudio 7 Pro System. The TaqMan Gene Expression Assay probes (ThermoFisher Scientific) used to assess changes in gene expression include *Acss2* (Assay ID: Mm00480101_m1), and *Rplp0* (control) (Assay ID: Mm00725448_s1). Samples were run as biological triplicates. Student’s *t*-tests were performed for statistical analyses.

### Intracellular cholesterol quantification:

Intracellular cholesterol quantification was performed using Cholesterol/Cholesterol Ester-Glo assay (Promega) according to manufacturer’s instructions. In short, cells were plated into two white opaque 96-well plate (PerkinElmer) and were cultured until ~80% confluency. One of those plates was used for normalization, the other one for cholesterol quantification. For normalization, cell viability was determined using CellTiter-Glo as described above. To measure cholesterol, media was removed and cells were washed twice with PBS. 50 μL of Cholesterol Lysis Solution was added to each well followed by gentle shaking of the plate and incubation for 30 minutes at 37°C. Then, 50 μL of Cholesterol Detection Reagent with or without esterase was added to each well and the plate was shaken for 30–60 seconds at a low rpm on a plate shaker. Cholesterol Detection Reagent without Cholesterol Esterase was used to measure free cholesterol, while with Cholesterol esterase was used to measure total cholesterol. Plates were then incubated for 1 hour at RT. Plates were read using EnVision 2102 Multilabel Reader (PerkinElmer). Cholesterol ester was calculated by subtracting free cholesterol from total cholesterol.

### Cellular fractionation:

Cellular fractionation was performed using NE-PER Nuclear and Cytoplasmic Extraction Kit according to manufacturer’s instructions. In short, cells were grown to about 80% confluence, harvested using TrypLE Express Enzyme (Gibco), and centrifuged at 500 × g for 5 minutes. After removing the supernatant, the pellet was resuspended in ice-cold CER I buffer, vortexed, and then incubated on ice for 10 minutes. Ice cold CER II was added to the mixture, vortexed, incubated on ice for 1 minute, and then centrifuged at 16,000 × g for 5 minutes. The supernatant (cytoplasmic fraction) was transferred to a new tube. The remaining pellet was resuspended in ice-cold NER buffer and incubated on ice for 40 minutes with intermittent vortex every 10 minutes for 15 seconds. Afterwards, the sample was centrifuged at maximum speed (16,000 × *g*) for 10 minutes. The supernatant (nuclear extract) was then transferred to a new tube. Protein quantification was performed using BCA protein assay and sample was diluted to similar concentrations in sample buffer (1X Laemmli Buffer, 5% 2-mercaptoethanol, BioRad). 20 μg of protein lysate was loaded onto 4–20% gradient Mini-PROTEAN TGX gel (BioRad) and western blotting was performed as described before. GAPDH was used as cytoplasmic marker and LAMIN A/C was used as nuclear marker.

### Glucose and acetate labeling:

Glucose and acetate incorporation into acetyl-CoA experiment was performed as follows. [U-^13^C]glucose and [1,2-^13^C]acetate incorporation into acetyl-CoAs were analyzed in cells incubated in DMEM without glucose, glutamine, or pyruvate (Gibco) with 10% dialyzed FBS (Gibco) in the presence of 5 mM [U-^13^C]glucose (Cambridge Isotope Laboratories) and 1 mM unlabeled acetate (ThermoScientific) or 5 mM unlabeled glucose (Sigma) and 5 mM [1,2-^13^C]acetate (Cambridge Isotope Laboratories) for 24 hours. Next day media was aspirated and 1 mL of ice-cold 10% Trichloroacetic acid (w/v) (Sigma) in water was added. Cells were scraped into a new tube and were frozen at −80°C. For relative acetyl-CoA determination, cells were incubated in the same conditions in the absence of labeled substrate. Cell volume and concentration were determined by TC20 Automated Cell Counter (BioRad).

### Acetyl-CoA measurements:

Acetyl-CoA lithium salt and 5-sulfosalicylic acid were from Sigma-Aldrich (P/N: A2181 and P/N: S2130, respectively). Optima^®^ LC/MS grade acetonitrile (ACN), formic acid, methanol, and water were purchased from Fisher Scientific. Oasis^®^ HLB 96-well elution plates (30 mg of sorbent) were purchased from Waters (P/N: WAT058951). Short-chain acyl-CoA internal standard (ISTD) was generated in yeast as previously described (PMID: 25572876).

Acyl-CoAs were analyzed by liquid chromatography-high-resolution mass spectrometry (LC-HRMS) as previously described^51^. 50 μL of short-chain acyl-CoA ISTD was added and then cell suspensions were sonicated with 5 × 0.5-second pulses at 50% intensity (Fisherbrand^™^ Sonic Dismembrator Model 120 with Qsonica CL-18 sonicator probe). Lysates were centrifuged at 17000 × g for 10 minutes at 4°C and clarified lysates were transferred to a deep-well 96-well plate for loading in a Tomtec Quadra4 liquid handling workstation. On the liquid handling workstation, lysates were applied to an Oasis HLB 96-well elution plate (30 mg of sorbent per well) pre-conditioned and equilibrated with 1 mL of methanol and 1 mL of water, respectively. After de-salting with 1 mL of water, acetyl-CoA was eluted into a deep-well 96-well plate using 1 mL of 25 mM ammonium acetate in methanol. Eluent was evaporated dried under nitrogen gas. The dried LC-HRMS samples were resuspended in 50 μL of 5% (w/v) sulfosalicylic acid in water. 5 μL injections of each sample were analyzed via LC-HRMS, using an an Ultimate 3000 quaternary ultra-high performance liquid chromatograph coupled with a Q Exactive Plus mass spectrometer (Thermo Scientific) as previously described^52^. A modified gradient using solvent A (5mM ammonium acetate in water), solvent B (5 mM ammonium acetate in 95:5 (v:v) acetonitrile: water) and solvent C (0.1% (v/v) formic acid in 80:20 (v:v) acetonitrile: water). Data was acquired using XCalibur 4.0 (Thermo Scientific), analyzed using Tracefinder 5.1 (Thermo Scientific), and corrected for normal isotopic distribution using FluxFix^53^.

### Acss2 CRISPR-mediated knockout:

Purified Cas9 nuclease and guide RNAs to target *Acss2* were ordered from Synthego. Cells were subcultured for 2 days before electroporation and were seeded so that they are about 80% confluent on the day of electroporation. About 100,000 cells were used per electroporation reaction. On the day of electroporation, cells were washed with PBS twice and then detached using TrypLE Express (Gibco). Cells were counted and diluted to appropriate concentration. Ribonucleoprotein (RNP) complexes were assembled at a ratio of 6:1 (sgRNA:Cas9) in 7 μL for a single reaction. The RNP solution was combined with 5 μL of cell suspension containing 100,000 cells and were electroporated in a 10 μL tip using Neon Transfection System (Thermo). Immediately after, electroporated cells were transferred to pre-warmed well of a 6 well plate and were cultured for 2–3 days. Confirmation of *Acss2* knock-out was performed by western blotting 72 hours after the electroporation. Single cell cloning was then performed and clones with successful knockouts were kept for subsequent analysis.

### In Vivo tumor syngeneic grafts:

Animal studies were conducted in accordance with the guidelines set forth by the Institutional Animal Care and Use Committee (Fox Chase Cancer Center institutional animal care and use committee (IACUC) #22–14). 5 × 10^5^ mouse colon epithelial cells were prepared in growth factor reduced Matrigel (Corning) 1:1 and injected into the right flank of 6- to 8-week-old male and female C57BL/6 mice. Mice were randomly split into the following arms based on the genetic background of cell line injected: *Apc*^*−/−*^*; Kras*^*WT/G12D*^*; Acss2*^*−/−*^*, Apc*^*−/−*^*; Kras*^*WT/G12V*^*; Acss2*^*−/−*^. There were at least 8 mice per group (4 males and 4 females). Tumors were monitored starting at approximately 150 mm^3^. Tumors were measured using a caliper every 3 days for 28 days in total. Tumor volume was calculated using the following formula: [(width)^2^ × (length)]/2. Student’s t-tests were performed for statistical analysis on tumor weights at the end of the experiment (day 28), P values ≤ 0.05 were considered significant.

### Apc^−/−^; Kras^WT/G12D^ vs. Apc^−/−^; Kras^WT/G12V^:

500,000 cells were subcutaneously injected in 1:1 Matrigel:PBS in the right flank of 6 week old C57BL/6 mice. Once tumors reached approximately 100 mm^3^, drug schedule commenced for 24 days. Tumors were measured every 3 days and volume was calculated by ((width^2^)*length)/2. Trametinib (Selleck S2673) was administered daily P.O.at 0.2 mg/kg dissolved in 0.5% hydroxypropylmethylcellulose, 0.2% Tween-80. ACSS2 inhibitor (Selleck S8588) was administered every 2 days via I.P. at 15 mg/kg and was dissolved in 5% DMSO, 40% PEG-300, 5% Tween-80, 50% H_2_O.

## Supplementary Material

1
