# Supplementary material for: KRAS mutation-selective requirement for ACSS2 in colorectal adenoma formation
Source: Res Sq. 2024 Feb 22:rs.3.rs-3931415. Preprint. [Version 1] doi: 10.21203/rs.3.rs-3931415/v1 (PMC10925460; doi:10.21203/rs.3.rs-3931415/v1)
Supplement: 1 [file NIHPPRS3931415V1-supplement-1.pdf]

Supplementary Figures

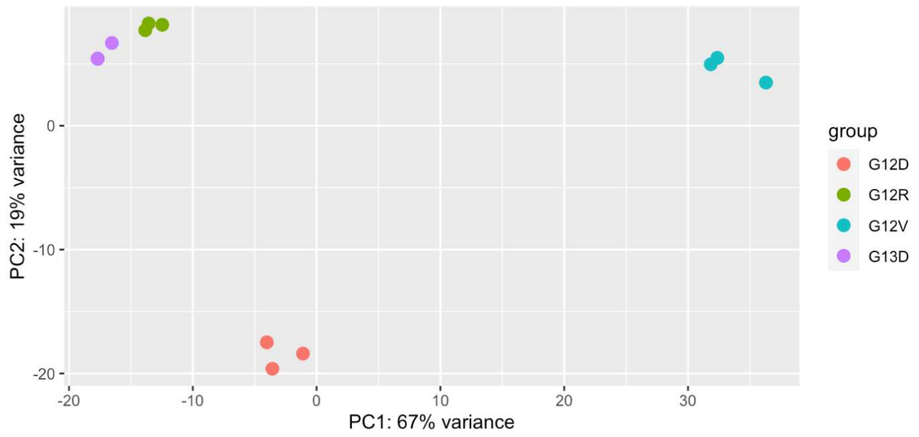

Supplementary Figure 1. Principal component analysis of *Kras*$^{WT/MUT}$ mouse colon epithelial cells.
Principal component analysis of genes with high variance in expression level, as determined by RNA sequencing.

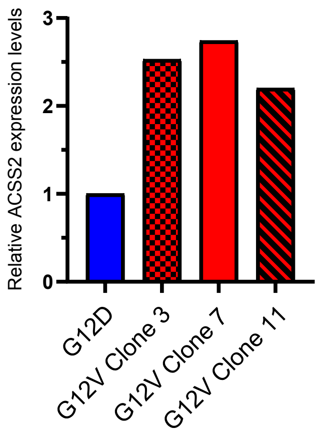

Supplementary Figure 2. Expression levels of *Acss2* in three G12V clones relative to G12D.

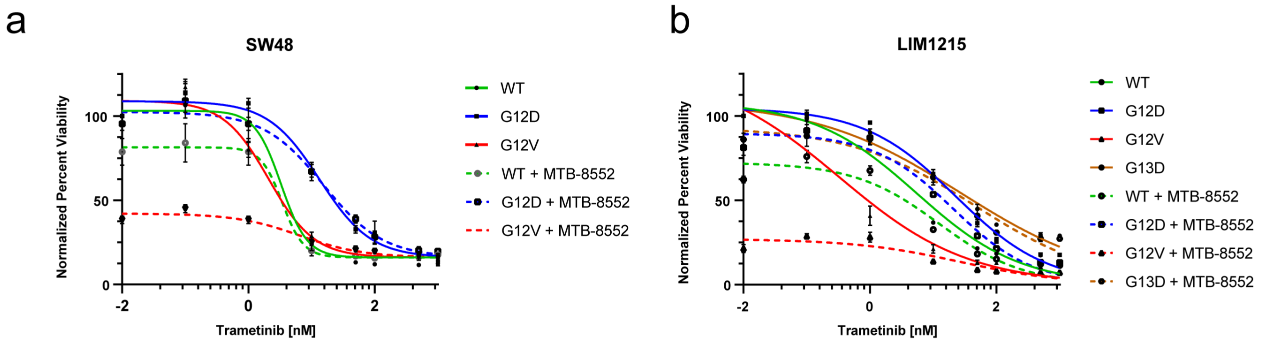

Supplementary Figure 3. Sensitivity of A) SW48 isogenic cell lines and B) SW48 isogenic cell lines to MEK inhibition alone or in combination with an ACSS2 inhibitor.
